# Management of deep caries

**DOI:** 10.1038/s41415-025-9350-7

**Published:** 2026-03-13

**Authors:** Alice R. Hamilton, David Ricketts

**Affiliations:** Dundee Dental Hospital and Research School, University of Dundee, United Kingdom

## Abstract

Deep dentinal caries, despite being preventable, is an ongoing widespread global issue. This review addresses deep carious lesions from aetiology through to management, and onto barriers in management implementation and ongoing research. Understanding the aetiology of dentinal caries and the histopathology of both carious lesions and the pulp-dentine complex reactions are crucial for providing the best clinical management, swinging the balance in favour of pulpal vitality. How caries removal techniques and terminology have changed over the past 100 years will also be explored together with current international guidelines and position statements on the management of deep carious lesions. However, despite this published guidance in support of more minimally invasive selective caries removal techniques, informed by decades of research, there remains a division among dental professionals globally on which technique should be routinely carried out for deep carious lesions. This paper briefly sets out some of the challenges associated with changing dental professionals' behaviour to align with implementation of the best available evidence, and how ongoing studies comparing the clinical and cost effectiveness of deep caries removal techniques and the environment for implementation are being led to strengthen the evidence base.

## Key points

- Deep caries management has evolved over the past century, following advancements in the understanding of the disease process, histological zones and modern materials.
- Evidence, including a Cochrane systematic review, supports selective caries removal techniques, as compared to complete removal.
- International clinical guidelines and position statements, which previously were often conflicting, are now aligning to support selective caries removal techniques
- Further studies are ongoing to inform on the best deep caries management strategies and the translation of this evidence into primary care.

## Introduction

In 2001, the second author to this publication wrote an article for the *British Dental Journal* (*BDJ*) entitled ‘Management of the deep carious lesion and the vital pulp-dentine complex'.^1^ Much had changed in the understanding of dental caries and the pulp since the teachings of G. V. Black nearly 100 years previously.^2^ Since 2001, interest in the management of caries, in particular, deep caries and maintaining a healthy vital pulp through ‘vital pulp therapies' has gained momentum. This paper aims to explore the breadth and depth of knowledge gained through research on this topic over the last 20 years or so and how this translates into clinical practice.

The focus of this publication will be on permanent teeth as this is where the long-term impact of appropriate management will be seen biologically, financially, in quality of life and impact on society.

## Global burden of disease

Following the widespread introduction of fluoridated toothpastes in the 1980s,^3^ the prevalence of caries was seen to dramatically decline in higher income countries.^4^ However, this initial optimism has not continued, with little change in caries prevalence over subsequent decades.^5^ Dental caries is still a problem in all age groups, with patients also living longer and retaining more teeth as they age.^4^ In fact, dental caries remains one of the most common non-communicable diseases worldwide, and the Global Burden of Disease Study 2015 found that untreated caries in permanent teeth was the most prevalent of all, impacting 2.5 billion people.^6^

While caries is an entirely preventable disease, if left unchecked, it can develop into extensive lesions with a profound effect on dental pulp health, potentially leading to reversible pulpitis, irreversible pulpitis, necrosis and eventually apical periodontitis and abscess formation. Although the prevalence of pulpitis is difficult to assess, especially as many teeth may be asymptomatic, it has been estimated globally that about half of the population have at least one tooth with apical periodontitis.^7^^,^^8^ Dental caries and its sequelae is undoubtedly the most common reason for carrying out endodontic treatment.^9^ These interrelated pathologies therefore require interrelated joined-up strategies to address their management in the future.

## Dental caries aetiology and histopathology

An understanding of the histopathology of caries and its close relationship to the pulp-dentine complex underpins caries clinical management and vital pulp therapies.

It has been estimated that the number of bacteria within the human body equates to the number of human cells.^10^ This huge number of microorganisms, or human microbiome, mostly exists in harmony with the human body, providing beneficial and protective effects. However, when this carefully balanced relationship breaks down, disease can occur, with caries a typical example.^11^

### Enamel caries

Oral bacteria that colonise the tooth surface (biofilm or dental plaque) have the potential, given the correct environment (sugar substrate, restricted disruption from oral function and oral hygiene measures, reduced salivary flow and limited topical fluoride) to produce sufficient acid to cause tooth tissue demineralisation. However, this process can be reversed by favourably controlling these environmental factors (caries prevention), leading to lesion arrest or remineralisation. This delicate dynamic balance takes place in the early stages of the disease process at a sub-clinical level, not seen by the naked eye. But if the environment tips this balance, favouring acid production and demineralisation, an enamel (white spot) lesion will eventually become visible clinically.

While our resident oral microorganisms are acquired from birth, dental caries is not an infectious disease caused by external pathogenic organisms passed from person-to-person. Instead, it is a non-communicable disease caused by a localised ‘ecological catastrophe'.^11^^,^^12^

### Early dentine caries

Continuation of this ‘ecological catastrophe' and imbalance in the biofilm on the surface of the tooth leads to demineralisation of the enamel in both density and depth. Eventually if the carious process is left unchecked, demineralisation reaches the enamel dentine junction (EDJ) and demineralisation of the dentine begins driven by acid produced by the biofilm microorganisms on the tooth surface diffusing into the tooth tissue. However, at this early dentine caries stage, modification of the oral environment through altered diet, oral hygiene and fluoride is still all that is needed to control the disease process.

### Moderate/deep dentine lesions

It is not until demineralisation extends well into the outer half of dentine that the over-lying enamel loses so much mineral that the surface breaks down and cavitation takes place.^13^

Frank cavitation of the tooth surface therefore occurs relatively late in the carious process, reflecting a position of no return where operative intervention is required. The microorganisms now colonise *en masse* within the cavity, protected from disruption. Further demineralisation and breakdown of dentine collagen from bacterially produced proteolytic enzymes can now spread more rapidly through the less mineralised dentine, leading to extensive dentine involvement or deep caries.

## What is deep caries?

**Fig. 1:**
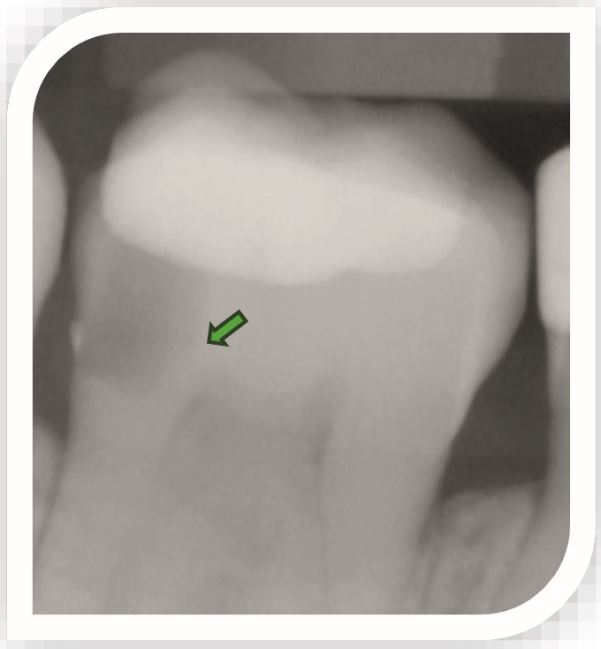
Deep caries distally in a restored lower molar. The arrow points to a dentine bridge between the carious lesion and the pulp
- Extremely deep caries – penetrating the entire thickness of the dentine with no clear zone of unaffected dentine separating the lesion from the pulp (Fig. 2).

Deep caries is defined as radiographically extending into the inner third or inner quarter of dentine with a risk of pulpal exposure.^14^ However, more recently Bjørndal *et al.* (2019)^15^ subdivided deep lesions further:

- Deep caries – extending radiographically into the pulpal quarter of dentine, but a clear zone of unaffected dentine separates the lesion from the pulp (Fig. 1)

**Fig. 2:**
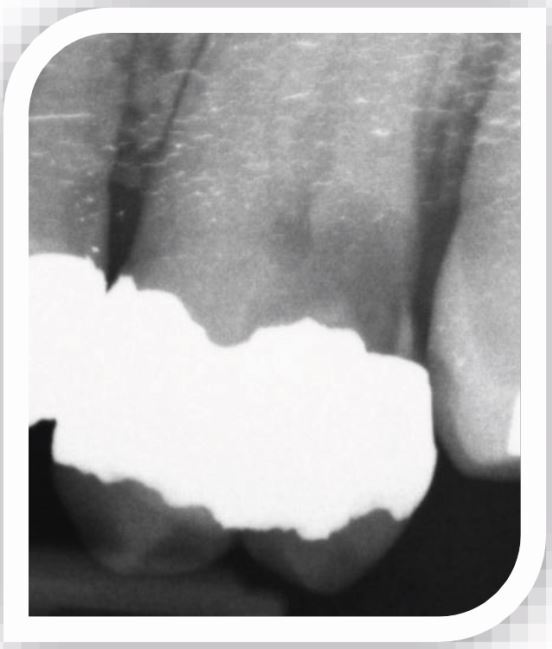
Extremely deep caries distally in a heavily restored upper molar. No dentine bridge can be seen between the carious lesion and the pulp

Visualising the extent or depth of a carious lesion from clinical examination alone is difficult and needs supplemented with radiographic examination. This paper focuses on the management of deep caries, but it is important to emphasise that interpretation of radiographic images can be subjective and is not a precise science due to the two-dimensional representation of a complex three-dimensional structure.

### Zones of dentine caries – histology and clinical significance

It has long been accepted that deeper caries is comprised histologically of two distinct zones; the outer zone (infected zone) and the inner zone (caries-affected zone).^16^^,^^17^^,^^18^^,^^19^^,^^20^ The infected (outer) zone is closest to the EDJ, and within this zone the dentine is severely demineralised, heavily infected with the oral microflora, and the collagen is broken down. This zone is not capable of remineralisation.

Acids and by-products from microorganisms within the infected outer zone diffuse towards the pulp, leading to dentine demineralisation ahead of this infected zone. This is the caries affected zone, where the dentine is not as severely demineralised and the collagen remains intact.

If the carious process is controlled by removing the infected (outer) zone, then the caries affected (inner) zone is minimally infected, capable of remineralisation and does not need removed. This caries affected inner zone is often stained and discoloured clinically;^21^ therefore, firm discoloured carious dentine pulpally is not a good guide for completing caries removal.

Unfortunately, the divide between these two histologically determined zones are not well-defined clinically. Early research suggested the use of various caries detector dyes to differentiate the zones, advising only the outer infected zone being stained with dye. However, more recent research has shown that the dyes are not as specific as originally thought, because they can also pick up the naturally less mineralised non-carious dentine toward the pulp and at the EDJ.^22^ Therefore, the use of these dyes cannot be recommended for general use to guide cavity preparation, as they can lead to over-preparation and greater risk to the pulp. Instead, the use of a spoon excavator pulpally has been shown in autofluorescent studies (the infected dentine marked through its autofluorescent signal) to be the most effective and efficient way of judiciously removing the outer infected zone and leaving the caries affected zone.^23^

### The vital pulp-dentine complex

Structurally, dentine and pulp are inextricably linked by the odontoblasts found within the pulpal periphery with their processes extending into the dentinal tubules, thus referred to as the pulp-dentine complex. Dentine itself is therefore regarded as a vital structure and the management of deep dentine caries should constitute the first line strategy in vital pulp treatments aimed at maintaining pulp vitality.^24^^,^^25^

Despite early enamel lesions reflecting a disease process taking place in the biofilm on the tooth surface, some studies have shown that the presence of microorganisms within these early non-cavitated lesions may even extend through to the EDJ or outer dentine.^26^^,^^27^ However, the significance of their presence in relation to caries progression is questionable as they may be in insufficient numbers to support lesion progression alone. The pulp-dentine complex also has an amazing ability to respond to dental caries and has been shown to respond to even these early stages of the disease process.^27^^,^^28^

Pulp-dentine complex reactions take place due to the released bacterial by-products including acids, together with bioactive molecules released from the demineralised and denatured dentine, diffusing toward the pulp, stimulating pulpal inflammatory and immune responses.^29^ The responses are complex but fundamentally depend on the severity of the stimulus; caries.

A low-grade stimulus, resulting from a slowly progressing lesion causes mild pulpal inflammation in the sub-odontoblast layer and the odontoblasts are upregulated to produce reactionary dentine similar to normal physiological dentine's tubular structure. More peritubular dentine may also be laid down within the dentinal tubules leading to tubular sclerosis. These responses aim to reduce the diffusion of injurious products toward the pulp.

With more severe stimulus, for example deeper and more rapidly progressing carious lesions, the inflammatory response progressively increases. The reactionary dentine may not be so ordered, with fewer dentinal tubules and cellular inclusions. At its most severe, death of the odontoblast layer will take place. Removal of the stimulus following appropriate caries management may lead to differentiation of pulpal stem cells into odontoblast-like cells and the formation of reparative dentine or calcific bridge.

Dentinogenesis of reactionary and reparative dentine have collectively been referred to as tertiary dentine formation and are not mutually exclusive to a specific lesion; reactionary dentine may dominate beneath the wider extremes of the lesion and reparative dentine in relation to the deepest aspect.^15^ The ability of the pulp-dentine complex to mount and maintain these protective responses depends upon the level of pulpal inflammation and continued pulpal health. However, evaluation of the actual pulp health is difficult from a patient history, especially as symptoms may be absent until the lesion is extremely deep, if presenting at all. Research showed that significant pathology of the pulp, including micro-abscess formation, did not occur until bacterial penetration of the carious dentine extended to within 1 mm of the pulp and only when reactionary dentine itself was invaded by bacteria that ‘pathosis of real consequence and of an irreversible nature was found'.^30^

A fine balance therefore exists between the carious process in deep lesions and pulp health, with the ultimate aim of operative dentistry to tip that balance in favour of the pulp.

## History of deep caries management strategies

### Non-selective caries removal/complete caries removal

It has historically been taught, and is still in some countries, that all traces of caries should be removed (including stained, caries-affected dentine) before restoration placement. It was thought that exposing the pulp and dealing with its consequences was better than the risk of leaving soft and infected caries.^2^ This has most often been referred to in the past as complete caries removal (updated terminology now ‘non-selective caries removal' [NSCR], see Table 1).

**Table 1:** New terminology used to describe various degrees of caries removal, the previously used terms and when to adopt each approach^14^

**New terminology**	**Old terminology equivalent**	**When to adopt**
Non-selective caries removal	Complete caries removal	No longer recommended for lesions of any depth
Selective caries removal to firm dentine* *includes firm or leathery dentine	Partial caries removal/indirect pulp cap, near complete, incomplete, or minimally invasive caries removal	Most carious lesions that are not thought to be deep
Selective caries removal to soft dentine	Partial caries removal, minimally invasive, incomplete, or ultraconservative caries removal	Deep carious lesions as defined by Bjørndal *et al.* (2019)^15^
Stepwise removal	Stepwise caries removal, stepwise excavation, two step caries removal	Deep carious lesions as defined by Bjørndal *et al.* (2019)^15^

However, concerns soon grew over this approach, and it was suggested that the exposed pulp was a doomed organ.^25^

### Direct pulp cap

Even careful caries removal in deep lesions, particularly in young patients with large pulps and pulp horns still has a significant risk of pulpal exposure.^31^ When the pulp tissue appears healthy and arrest of haemorrhoage readily achieved, the first line vital pulp therapy for many would be a direct pulp cap, due to its relative ease of application, minimal invasiveness and reduced cost compared to endodontic treatment. Retrospective studies carried out in dental schools observing the long-term success rate of direct pulp caps after carious exposure showed that after 3–5 years only about one-third (33–37%)^32^^,^^33^ were successful, and after five years only 13% were successful.^33^ Improvements in techniques and dental materials has seen a dramatic improvement in success rates with a systematic review showing 84–86% success after 2–3 years using hydraulic calcium silicate cements as the capping agent.^34^ However, the included studies often had small sample sizes and the authors concluded that the studies were of fair to poor quality with a high risk of bias.

With such conflicting and inconclusive evidence, avoidance of carious exposure would be the ideal goal and various caries removal strategies have been described to achieve this.

### Indirect pulp cap

As a result, the indirect pulp cap was proposed and is still advocated today as part of the vital pulp therapy armamentarium. The indirect pulp cap has been described for deep lesions as ‘almost completely' removing ‘demineralised and discoloured dentine leaving a thin layer of residual caries' over the pulp which ‘if removed with an excavator will certainly expose the pulp'.^35^ This traditionally was lined with setting calcium hydroxide but has been superseded with the superior hydraulic tricalcium silicate cements.

Reflecting on the indirect pulp capping technique and the now widely accepted selective caries removal of only the outer zone of caries and leaving the inner stained zone (as described in the following section), there appears to be little difference if any. As such perhaps the term ‘indirect pulp cap' for the management of deep caries is now a redundant term and should be relegated to the history books?

### Minimally invasive deep caries management

Historically, the driving force behind the complete removal of caries had been the need to remove all infected carious dentine, to ensure the carious process could not continue beneath the restoration, and remove demineralised softened dentine so that the restoration could be packed against a sound tooth surface. However, there is now a wealth of evidence from numerous types of study which support that not all soft and infected carious dentine needs to be removed. In permanent teeth, researchers have investigated the following.

#### For occlusal dentine lesions

- Placing resin fissure sealants to seal in dentine lesions that are radiographically visible. Such lesions have been shown to extend through to the middle third of dentine and likely to be heavily infected^36^^,^^37^^,^^38^^,^^39^^,^^40^^,^^41^^,^^42^^,^^43^
- Ultraconservative caries removal, where the enamel caries at the entrance to the fissure is removed, but without any dentine caries being removed, and restored with a composite restoration.^44^

#### For occlusal and approximal extensive coronal lesions

- Stepwise excavation (updated terminology now ‘stepwise removal' [SW], see Table 1). Two-stage caries removal. First visit: access to caries and periphery of cavity rendered totally caries/stain free, with pulpal caries removed sufficiently to place a restoration of adequate thickness leaving frankly soft caries. Cavity lined (optional) and restored with an adhesive (glass ionomer or composite resin) provisional restoration and left for 6–12 months. Second visit: provisional restoration removed, residual caries excavated and definitive restoration placed^45^
- Partial caries removal (updated terminology now ‘selective caries removal' [SCR] see Table 1). Essentially, this is the first stage of SW excavation and the placement of a definitive restoration sealing the residual dentine caries over the pulp without re-entering.^46^^,^^47^

## Re-evaluating terminology and clinical management – International Caries Consensus Collaboration

With increasing clinical and research interest in the ‘management of deep caries' since the 2001 *BDJ* publication, there has been a concomitant rise in the number of publications and terms used in relation to caries removal. This has been the source of some confusion with different terms being used to describe the same technique.^14^^,^^48^ In addition, a carious lesion is continuous and progressive through dentine with no definitive boundaries to delineate between hard, firm, leathery or soft dentine. This terminology for dentine hardness has been used widely in the literature; however, there are no convenient aids to assist in differentiating between these clinically. There is little surprise that assessment of carious dentine's hardness is very subjective from one practitioner to another. One dentist's hard, may be another's leathery or even soft. This adds confusion to the flavours of caries removal techniques outlined in the previous sections.

To address some of these issues and reach a consensus, the International Caries Consensus Collaboration (ICCC) was formed and produced recommendations on terminology^14^ and operative management of carious tissue removal.^48^ Table 1 summarises the updated international consensus on terminology for operative procedures, including when to adopt them, and Table 2 shows the definitions for the clinical presentation of dentine.

**Table 2:** The clinical presentation of carious dentine and definition^14^

**Clinical presentation of dentine**	**Definition**
Soft	‘Will deform when a hard instrument is pressed onto it and can be easily scooped up (e.g., with a sharp hand excavator) with little force being required'
Leathery	‘Although the dentine does not deform when an instrument is pressed onto it, leathery dentine can still be easily lifted without much force being required'
Firm	‘Firm dentine is physically resistant to hand excavation, and some pressure needs to be exerted through an instrument to lift it'
Hard	‘A pushing force needs to be used with a hard instrument to engage the dentine, and only a sharp cutting edge or a bur will lift it. A scratchy sound or ‘*cridentinaire'* can be heard when a straight probe is taken across the dentine'

While various terms have been used to describe operative procedures during cavity preparation, ‘caries removal' was preferred to avoid confusion with other caries removal techniques such as excavation (e.g., with a spoon excavator).

New terms were derived to avoid terms such as ‘complete', ‘partial' or ‘incomplete' caries removal, which brought with them negative connotations, implying that the latter was in some way sub-standard.

Partial caries removal covered a spectrum of caries removal endpoints and was therefore redefined as SCR, and further split into two separate definitions to reflect different intended operational endpoints (either to soft or firm dentine over the pulp). Historically, many studies did not make this distinction, but going forward, it is important to define the intended endpoints, as leaving only firm-stained dentine may carry an entirely different risk to the pulp than leaving soft dentine.

## Evidence for minimally invasive techniques

The numerous studies published looking at more minimally invasive caries removal techniques have the same underlying theme of sealing soft, infected carious dentine into permanent teeth and the highest quality available evidence has been evaluated in three consecutively updated Cochrane systematic reviews, with the latest published in 2021.^31^^,^^49^^,^^50^ Salient research findings can be summarised as:

- Sealed dentine caries with a resin restoration through ultraconservative caries removal, has been associated with no evidence of lesion progression over time, no significant loss or deterioration of restorations, no reports of signs or symptoms of pulp pathology with one study having ten-year follow-up^44^
- Sealing dentine caries leads to changes in colour and consistency of the dentine consistent with an arrested lesion^45^^,^^51^^,^^52^^,^^53^^,^^54^^,^^55^
- Once sealed, a reduction of viable organisms occurs over time, due to restriction of sugar substrate from the diet/oral cavity^51^^,^^52^^,^^54^^,^^55^^,^^56^^,^^57^
- Once sealed, a reduction in the diversity of organisms contributing to the microbial load.^57^ Only bacteria capable of breaking down glycoproteins from pulpal tissue fluids survive, and these are not associated with active lesions
- Meta-analysis in one Cochrane systematic reviews showed a significant reduction in risk of pulpal exposure with less invasive techniques.^31^ SW can lead to a 49% reduction in risk of pulpal exposure in permanent teeth and 69% in primary teeth, while SCR leaving softened dentine over the pulp in a one-stage technique can result in a 77% reduction in risk of pulp exposure in primary and permanent teeth combined
- The three studies^46^^,^^58^^,^^59^ on permanent teeth included in the 2021 Cochrane systematic review^50^ provided evidence that SCR leaving softened dentine over the pulp reduces failure compared to SW over a 12–60-month follow-up. This may be due to the risk with SW that, on re-entering, there is additional trauma to the pulp-dentine complex
- Stressed pulp syndrome has long been recognised, in which the pulp-dentine complex repairs following operative trauma but has a diminished ability to repair following repeated trauma.^60^ Therefore, re-entering with SW carries with it the risk of further trauma to the pulp-dentine complex and, albeit much reduced, the risk of pulpal exposure.

However, although the ever-growing evidence base supports more minimally interventive techniques, studies often have small sample sizes, leading to a high risk of bias. Clinical trials are also often carried out in a secondary care setting and do not always measure the ‘real-life' application of the techniques in the wider primary care setting. Further high-quality studies are required to strengthen this evidence base.

One ongoing clinical trial – the Selective Caries Removal in Permanent Teeth (SCRiPT) study – is a multi-centre, pragmatic, randomised control trial currently being carried out in primary care dental practices across Scotland and England.^61^ The National Institute for Health and Care Research Health Technology Assessment (HTA) programme has commissioned and funded a collaborative team from universities across the UK to carry out this study (trial number: HTA 17/127/07/ISRCTN76503940), which compares the clinical and cost effectiveness of SCR (with the intention of leaving soft dentine over the pulp), compared to complete (NSCR) or near complete caries removal (SCR to firm/leathery dentine). The SCRiPT study is due to be completed in 2026 with an average follow-up of three years and results are expected to have an impact on the provision of treatment for deep carious lesions globally by adding strength to the evidence base.

## But what do dentists do globally?

Since the 2001 *BDJ* publication^1^ and 2016 ICCC recommendations^14^^,^^48^ many studies have shown that there is still little agreement among dentists internationally on how to best manage deep carious lesions.

Edwards *et al.* showed this divide in the UK, with a survey conducted in 2021, where equal numbers of primary care dental professionals reported using NSCR and SCR, with 41.4% each.^62^ However, more recent unpublished survey data have suggested a further shift towards conservative techniques, with only 28% of UK dentists reporting NSCR as their typical technique in 2023.^63^ A similar trend was shown in the USA, with reported implementation of SCR by only around 22% and 34% of dentists in 2006 and 2010, respectively.^64^^,^^65^ However, in 2022, this increased to 62.4% of dentists reporting to use SCR more than 50% of the time for asymptomatic deep carious teeth, decreasing to 49.3% for the management of symptomatic teeth.^66^ Alternatively, dentists in Norway and Germany report SW as their most used deep caries removal technique, with 84% and 48%, respectively, compared to dentists in France where the majority favoured NSCR.^67^ Dentists in Spain were one of the most polarised groups with only 8% of dentists reporting they would carry out SCR for a tooth with reversible pulpitis and 88% choosing NSCR in one or two stages.^68^ The question arises as to why there is such divided behaviour in regards to deep caries removal bearing in mind the aforementioned evidence supporting minimally invasive techniques over NSCR.

Studies have shown that dentistry has many complexities which may influence behaviour such as individual clinicians' knowledge, beliefs or fear of failure with change, social influencers (including fear of litigation or judgement from peers), varying undergraduate curriculums, access to resources and remuneration.^69^^,^^70^^,^^71^^,^^72^ Understanding this landscape is essential for navigating implementation of the best quality of evidence into clinical practice. However, changing behaviour is known to be extremely challenging and it is not as simple as publishing research or clinical guidelines and expecting them to be applied into practice. To tackle this, robust evidence-based theory-grounded behaviour change approaches must be used.

Further research by the lead author is being undertaken into the barriers and facilitators to changing behaviours regarding the implementation of the highest quality of evidence in relation to caries removal techniques.^63^ Translational studies are also required to measure the impact and efficacy of any applied interventions to reduce resource and research wastage, in addition to improving health outcomes, patient quality of life, and improved sustainability with a reduced restorative cycle.

## Current clinical guidelines and position statements

Despite this worldwide conflict of opinions on the best management for deep carious lesions among dental professionals, there are a number of guidelines and position statements published internationally, as summarised in Table 3.^73^^,^^74^^,^^75^

**Table 3:** The main guidelines and position statements currently available for the management of deep carious lesions in permanent teeth

**Guideline/position statement**	**Scope**	**Summary of main recommendations for deep caries management in permanent teeth**	**Further comments**
International Caries Consensus Collaboration (ICCC): Recommendations on Carious Tissue Removal (2016)^14^	Consensus for the management of carious lesions in primary and permanent teeth. Developed by a group of 21 clinical experts from 12 countries, with expertise in cariology, operative dentistry, biomaterials science, clinical trials, systematic reviews and guideline development	Recommendations for deep carious lesions into the pulpal third or quarter with vital pulps: SCR leaving soft dentine over the pulp (strongly recommended). SW considered a possible alternative. Advise against: Cavity disinfection due to a lack of evidence of benefit Use of caries detector dyes as these may lead to over-treatment. Cavity linings: Not recommended for improving cavity seal but may be considered to prevent monomer penetration during composite placement or thermal injury to the pulp with amalgam restorations	One of the most detailed consensus statements relating to caries removal strategies as they advise on the consistency of dentine to be left over the pulp with SCR – often left to the reader's discretion in other guidance/position statements. This guidance advises NSCR is no longer recommended as for cavitated lesions (considered over-treatment)
Scottish Dental Clinical Effectiveness Programme (SDCEP): Prevention and Management of Dental Caries in Children Guidance (2025)^73^ (Third edition) *National Institute for Health and Care Excellence (NICE) accredited	Clinical practice guidance for prevention and management of caries in children, covering primary and permanent teeth	Recommendations for vital posterior permanent teeth with deep carious lesions: SCR leaving soft dentine over the pulp if a dentine bridge is visible between the carious lesion and the pulp radiographically Consider SCR or pulpotomy for extremely deep lesions where no dentine bridge is visible between the carious lesion and the pulp radiographically	This guidance has a dedicated website containing useful clinical resources, including: Flowchart and table to aid clinical decision making – located in the ‘Caries management for permanent teeth' section Step-by-step clinical guides for SCR and pulpotomy techniques in permanent teeth – located in the ‘Dental techniques' section
European Society of Endodontology (ESE) position statement: Management of Deep Caries and the Exposed Pulp (2019)^74^	Consensus from an expert committee setting out guidance on diagnosis, classification of deep caries and pulpal disease, and their management	For asymptomatic permanent teeth with deep caries or showing signs of reversible pulpitis: SCR or SW is recommended, ideally with: Rubber dam Magnification Hydraulic calcium silicate cement or glass ionomer cement over the deep dentine. Where a carious pulpal exposure occurs: Isolation with rubber dam, sterile instruments used, and the tooth disinfected before vital pulp therapy with either: Pulp cap or partial pulpotomy using a hydraulic calcium silicate cement. Progressing to: Full pulpotomy – if partial irreversible pulpitis Pulpectomy – if more fully irreversibly inflamed	This position statement divides deep caries into the accepted ‘deep' and ‘extremely deep' categories described earlier in this paper.^15^ The remaining dentine recommended to be left over the pulp in SCR is not discussed (whether firm, leathery or soft following cavity preparation)
American Dental Association (ADA): Evidence-based Clinical Practice Guideline on Restorative Treatments for Caries Lesions (2023)^75^	Clinical practice guidelines developed for USA with recommendations for the management of caries in primary and permanent teeth	Recommendation for pulpal dentine for the management of deep carious lesions in permanent teeth SCR to firm or soft, preferable over NSCR and SW techniques (recommended with low certainty and advised further research)	These guidelines presented a dynamic shift to align more with European guidance and international consensus, with a higher emphasis on more minimally invasive caries removal techniques and away from its more traditional preference for NSCR
NSCR, non-selective caries removal; SCR, selective caries removal; SW, stepwise removal			

The aim of clinical guidelines and position statements are to provide clear and concise recommendations and expert consensus from the best available evidence. However, it is not as simple as publishing a guideline and expecting clinicians to implement it into their clinical practice. Many dentists are balancing a busy work life and may not have time or opportunity to seek out, navigate, and digest available published guidance. Unpublished research from a questionnaire assessing UK primary care dentists' management of deep carious lesions in permanent teeth confirmed that many practitioners are not aware of guidelines, with only 15% of the 410 surveyed dentists able to provide knowledge of a guideline on caries management with the most known guidance being published by the Scottish Dental Clinical Effectiveness Programme, closely followed by the European Society of Endodontology 2019 position statement.^63^

Further challenges in implementing evidence-based caries removal techniques have arisen due to previously published international guidelines often having conflicting recommendations, with some promoting NSCR, while others promoting SCR or SW. However, this has recently changed, as throughout almost all of the currently published key guidelines and position statements internationally, there is now alignment with a strong global theme promoting more minimally invasive caries removal techniques (Table 3). The American Association of Endodontists Position Statement on Vital Pulp Therapy (2021) may be considered an outlier, advocating for a less conservative approach, stating *‘*complete caries removal is essential to eliminate infected tissues and visualise pulp tissue conditions under magnification when pulpal exposures occur'.^76^ However, this statement may add further to the confusion, as it is not clear whether it advocates NSCR for the management of all deep lesions, or only those with pulpal exposure. A further challenge in primary care is that magnification may not be routinely available to the clinician, and the statement also promotes the use of caries detector dyes, which as mentioned previously, may lead to overpreparation and removal of healthy tooth tissue.^22^

Throughout all the guidelines and position statements, it is agreed that it is essential, irrespective of which caries removal approach is used pulpally, that the dentine at the periphery of the cavity (the EDJ or outer 2 mm of dentine if extending onto the root) should be hard with similar tactile characteristics to sound dentine. This allows good adaptation and bond of the restorative material to cavity, crucially creating a good peripheral seal.

## Conclusion

Over the past century, there have been advances in the understanding of deep carious lesions and the pulp at an aetiological, histopathological, and clinical level, which has in turn been driven by improvements in materials and techniques. The development of more minimally invasive selective caries removal techniques, such as SCR, manages the carious process from a biological perspective, with a good peripheral seal on sound dentine crucial for success. This leads to a reduction in the number and diversity of viable microorganisms within the sealed caries and prevention of further bacterial ingress. Balance is tipped in favour of the pulp by arrest of the carious process to allow resolution of pulpal inflammation and promote tubular sclerosis and tertiary dentine formation. The strength of evidence in support of these techniques is being further strengthened by ongoing studies, which will help to inform future development of guidelines, undergraduate and postgraduate education and other theory-grounded behavioural change interventions to unite and empower dental professionals to apply the best evidence-based practices regarding deep caries management.
